# Preparation, characterization, and biodistribution of glutathione PEGylated nanoliposomal doxorubicin for brain drug delivery with a post-insertion approach

**DOI:** 10.22038/IJBMS.2022.60306.13369

**Published:** 2022-03

**Authors:** Amin Mehrabian, Roghayyeh Vakili-Ghartavol, Mohammad Mashreghi, Sara Shokooh Saremi, Ali Badiee, Leila Arabi, Seyedeh Hoda Alavizadeh, Seyedeh Alia Moosavian, Mahmoud Reza Jaafari

**Affiliations:** 1 Department of Pharmaceutical Nanotechnology, School of Pharmacy, Mashhad University of Medical Sciences, Mashhad, Iran; 2 Nanotechnology Research Center, Pharmaceutical Technology Institute, Mashhad University of Medical Sciences, Mashhad, Iran; 3 Warwick Medical School, University of Warwick, Coventry, UK; 4 Department of Medical Nanotechnology, Shiraz University of Medical Sciences, Shiraz, Iran; 5 Biosun Pharmed Pharmaceuticals Company, Tehran, Iran; 6 Biotechnology Research Center, School of Pharmacy, Mashhad University of Medical Sciences, Mashhad, Iran

**Keywords:** Biodistribution, Blood-brain barrier, Brain drug delivery, Glutathione, Liposome, Post-insertion

## Abstract

**Objective(s):**

Brain cancer treatments have mainly failed due to their inability to cross the blood-brain barrier. Several studies have confirmed the presence of glutathione (GSH) receptors on BBB’s surface, as a result, products like 2B3-101, which contain over 5% pre-inserted GSH PEGylated liposomal doxorubicin, are being tested in clinical trials. Here we conducted the PEGylated nanoliposomal doxorubicin particles that are covalently attached to the glutathione using the post-insertion technique. Compared with the pre-insertion approach, the post-insertion method is notably simpler, faster, and more cost-effective, making it ideal for large-scale pharmaceutical manufacturing.

**Materials and Methods:**

The ligands of the DSPE PEG(2000) Maleimide-GSH were introduced in the amounts of 25, 50, 100, 200, and 400 on the available Caelyx. Following physicochemical evaluations, animal experiments such as biodistribution, fluorescence microscopy, and pharmacokinetics were done.

**Results:**

In comparison with Caelyx, the 200L and 400L treatment arms were the most promising formulations. We showed that nanocarriers containing 40 times fewer GSH micelles than 2B3-101 significantly increased blood-brain barrier penetrance. Due to the expressed GSH receptors on tissues as an endogenous antioxidant, doxorubicin will likely concentrate in the liver, spleen, heart, and lung in comparison with Caelyx, according to other tissue analyses.

**Conclusion:**

The post-insertion technique was found a successful approach with more pharmaceutical aspects for large-scale production. Moreover, further investigations are highly recommended to determine the efficacy of 5% post-inserted GSH targeted nanoliposomes versus 2B3-101 as a similar formulation with a different preparation method.

## Introduction

Brain cancer is among the most aggressive and devastating types of malignancies. Statistically, it includes almost 90% of primary tumors in the central nervous system (CNS). The American cancer society has estimated 23,890 newly diagnosed cases and 18,020 deaths as a result of brain tumors in 2020. A relative 5-year survival rate of 32.6% has been reported between 2010-2016 for brain cancer. The survival rates considerably depend on several factors including age, sex, and tumor types (1, 2). Surgery, radiation, and chemotherapy are frequently used in clinical management, either alone or in combination (3). Even though tumor resection surgery has improved overall survival rates and other related outcomes, it is not as effective as when it is combined with other available treatments. Tumor heterogeneity and complexity plus the visual bias at the tumor margins during tumor removal are just a few of the complications impacting the efficacy of this procedure. Moreover, patients choose alternate options due to the surgery’s severe nature (4-6). Recently, radiotherapy both independently or in combination with other treatments has become one of the main approaches for the treatment of brain tumors and has been successful in prolonging patients’ survival rates. However, even with the most cutting-edge radiation devices, radiotherapy processes are not without flaws, and as a result, radiotherapy-related side effects lead to radiation restrictions (7-10). Chemotherapy is the least aggressive and most efficient choice that has been developed and improved over the years. However, the adverse effects, poor bioavailability, and limited efficacy are still challenging issues and further improvements seem necessary (11-13). Recently, nano-formulations have demonstrated promising preclinical and clinical results in the treatment of cancer, especially brain cancer. An encouraging example of nanocarrier products in the clinic is Caelyx^®^ or PEGylated liposomal doxorubicin. It has been widely and successfully utilized to treat a variety of cancers. The liposomal nanostructure not only has diminished the previously reported doxorubicin toxicities (such as cardiac, hepatic, skin, neuronal toxicities, etc.), but it also has improved and enhanced the efficacy and bioavailability (14-18) making it a better approach than the conventional formulations with the hydrophilic PEGylated shell and nano-sized structure (approximately 100 nm). Additionally, the flexibility in physicochemical modifications and preparation of liposomal nanostructures has enhanced the efficacy and dramatically decreased the adverse effect (19-22). The inflammation caused by malignancies enhanced permeation and retention (EPR), a well-known mechanism for cancer medication nano delivery systems (23, 24). Clinical investigations, on the other hand, show that nanoparticles, particularly in brain tumors, have not been able to achieve therapeutic quantities in the tumor site. Studies have shown that the EPR effect is highly dependent on the nature and location of the tumor. The situation with brain tumors is more complicated since the presence of the blood-brain tumor barrier adds an additional limiting factor in reaching the tumor interstitium (25-28). Therefore, the EPR effect has the minimum contribution in tumor site accumulation in the initial stages of brain tumors (29, 30).

Despite many advances in the characteristics of liposomal nanoparticles, crossing the blood-brain barrier (BBB) remains a challenge with available formulations (31-33). A successful drug delivery approach to the brain has to circumvent several BBB limitations. The BBB efflux transporters minimize therapeutics entrance resulting in a phenomenon called pharmacoresistance. The paracellular pathway is limited due to the tight junctions among BBB cells. These connections make the intercellular gaps less than 1 nm, which is only permeable to water and a few trace elements. Among different strategies, the intracellular pathway represents the most promising approach for lipophilic structures with less than 500Da molecular weight. Most of the essential compounds for the central nervous system pass through the BBB via the receptors on the BBB outer layer. For example, glucose, insulin, and transferrin get through the BBB by receptor-mediated transcytosis. Previous studies proposed the efficiency of nano-carriers in the anti-cancer agents’ brain delivery can be improved by active targeting as a result of increasing receptor-mediated uptake. The aforementioned receptors have been the target of numerous nanocarrier investigations so far but none have been successful in the clinic (34-43), although there is still a need to investigate formulations that could make their way to the clinical phases. 

Glutathione (GSH) is an endogenous tripeptide with a negative charge that has shown a neuroprotective role in the CNS due to the anti-oxidant effects (44, 45). Previous publications have shown GSH receptor expression on the surface of the BBB (46-48).

Glutathione PEGylated nanoliposomes have well been used as a brain drug delivery platform. 2B3-101, which has finished the phase I/IIA clinical trial, is glutathione coated Doxil^®^/Caelyx^®^ which is prepared by incorporation of DSPE-PEG(2000) Maleimide-GSH into Caelyx’s structure (49-51).

2B3-101 is prepared by GSH anchored PEG micelles into the liposomal structure with conventional lipids followed by remote loading of doxorubicin (52-55). The GSH targeting ligands are incorporated into the liposomal bilayer on both the inner and outer surfaces using the pre-insertion method. It’s worth noting that lipophilicity of targeted ligands determines their orientation toward the liposomal bilayer. Even though this method, as a modification strategy of nanostructures, results in the least amount of therapeutic leakage during the production procedure, the orientation of targeting ligands towards the inner surface of liposomes, on the other hand, causes increased viscosity, extrusion problems, and sterical instability (56-61). Furthermore, due to unnecessary positioning of targeting ligands into the inner liposomal bilayer, the pre-insertion process is not economically or technically ideal for valuable targeting ligands. There is an unmet demand for a more selective strategy to load valuable and expensive targeted ligands just on the outer surface. The post-insertion technique offers a simpler, faster, and more cost-effective approach making it desirable for large-scale pharmaceutical manufacturing. In this method, the ligand-coupled PEG-lipid derivatives are applied to the liposomes from a micelle phase ((57, 59, 60, 62-64). Previously, we demonstrated the high efficiency and reproducibility of the post-insertion method in the preparation of actively targeted liposomal Dox (65, 66). In a temperature- and time-dependent manner, the micellar DSPE-PEG(2000) Maleimide-GSH complex has been incorporated into the PEGylated liposomal doxorubicin structure. The optimum time and temperature have been obtained through outputs of previous experiments (62, 67).

This study aims to demonstrate that glutathione decorated PEGylated liposomal doxorubicin distribution to the brain is approachable successfully through utilizing the post-insertion technique to which the GSH targeting ligands are incorporated into the commercially available Caelyx^®^. The post-insertion methodology produces robust and highly reproducible products in comparison with the pre-insertion method which has been used by Gaillard *et al*. in similar studies in tumor-induced rodents. Moreover, we showed that in comparison with 2B3-101, 40 times lower levels of GSH micelles have significantly increased the penetrance through the blood-brain barrier, establishing the minimum required number of ligands for effective delivery to the brain. (68, 69).

## Materials and Methods

***Materials***

Methoxypolyetheleneglycol (M.W. 2000) distearylphosphatidylethanolamine (mPEG2000-DSPE) was prepared from Lipoid (Ludwigshafen, Germany). Doxorubicin hydrochloride (Dox) was purchased from Sigma–Aldrich (St. Louis, MO, USA). Maleimide PEG2000 di stearoyl phosphatidylethanolamine or DSPE-PEG(2000) Maleimide was purchased from Avantipolar (Alabaster, AL, USA).  γ-L-Glutamyl-L-cysteinyl-glycine (L-Glutathione) and doxorubicin hydrochloride were purchased from Merck and Sigma-Aldrich (St. Louis, MO, USA), respectively. Isopropanol was purchased from Merck for acidified isopropyl alcohol preparation. The 90% isopropanol/0.075 M HCl was arranged by addition of 2.5 ml water and 7.5 ml HCl 1 M to the 90 ml isopropanol. Commercially available Caelyx^®^ was prepared from BehestanDarou Company (Tehran, Iran). Other reagents and solvents were used as a chemical grade. Acidified isopropyl alcohol (90% isopropanol/0.075 M HCl) was prepared by adding 2.5 ml water and 7.5 ml HCl 1 M to 90 ml isopropanol (Merck, Darmstadt, Germany). 

***GSH-PEG2000 preparation***

In order to covalently link the GSH peptide to the DSPE-PEG(2000) Maleimide, the peptide was dissolved in dimethyl sulfoxide and added to the DSPE-PEG(2000) Maleimide chloroform solution. The peptide to maleimide molar ratio was 1.2:1 and the DMSO to chloroform volumetric ratio was 1:1. They were mixed continuously for 48 hr at 37 °C for the mentioned reaction. In the end, with a rotary evaporator (Heidolph, Germany), the solvents were removed and were freeze-dried (VD-800F, Taitech, Japan) (70).

***GSH-PEG2000 evaluation***

The freeze-dried product was dissolved in ammonium sulfate and analyzed by thin-layer chromatography (TLC). The peptide-lipid conjugate was monitored against the TLC plate with the ratio of 45/9/1 chloroform/methanol/water mobile phase and iodine vapor exposure.

Moreover, the peptide reaction with the PEG was evaluated with reverse-phase liquid chromatography (Shimadzu, Japan). The complex was assessed in an isocratic gradient condition with the 0.001 phosphoric acids HPLC grade water as the mobile phase. For the procedure validation, free GSHs were added to the final product without any maleimide reactive groups and were treated the same as others.

***Post-inserted formulation preparation***

The GSH-PEG prepared micelles were incubated with Caelyx^®^ at 60 °C for 1 hr while stirring gently. Then, their phosphate content was determined by the Bartlette assay. Consequently, the GSH-Caelyx^®^ post-inserted formulations were prepared by insertion of 25, 50, 100, 200, and 400 ligands on the liposomal surface. The number of peptide molecules per liposome was calculated based on the following parameters: Caelyx^®^ phospholipids concentration, liposomal average size and per liposome lipid molecules for their average size, liposomal numbers per each milliliter, total peptide content, peptide molecules per each milliliter of peptide-micelles, and peptide number aimed to put on each liposome surface (65, 67, 71, 72). The liposomes were analyzed based on the fluorescent of Dox to estimate drug content post-reaction. The fluorimetry device was used for the mentioned purpose.

***Liposomal characterization***

Produced 25, 50, 100, 200, and 400 ligand nanoliposomes were characterized based on size, zeta-potential, and polydispersity index (PDI) by a Dynamic Light Scattering instrument (Nano-ZS; Malvern, UK) (73). Dox encapsulation efficiency was measured before and after post-insertion. As indicated in the materials section, the PEGylated liposomal doxorubicin utilized was commercially available (2 mg/ml) and was considered 100%. The formulations before and after the post-insertion were evaluated as follows. The dialysis method was used to remove the post-insertion procedure’s released doxorubicin. In brief, using a 12-kDa molecular weight cut-off dialysis membrane, the free doxorubicin was removed from liposomes by dialyzing against dextrose/histidine buffer (pH 6.5). In order to determine Dox concentration, aliquots of preparations (20 µl) were dissolved in acidified isopropyl alcohol 90% (1800 µl) +180 µl dextrose, vortexed and incubated for 10 min at 70 °C. The Dox concentration was measured by spectrofluorometer (ex: 470/em: 590) (Shimadzu RF5000U, Japan). The encapsulated Dox percentage was determined by the following formula: % Dox encapsulated = ([Dox concentration after post-insertion]/[ Dox concentration before post-insertion]) × 100.

Release studies were conducted by the dialysis method in three different media with a pH of 7.4 (PBS), 6.5 (dextrose histidine), and 5.5 (dextrose succinate) (74, 75). The dialysis bags with formulations content were put separately in mentioned buffers and incubated at 37 °C overnight. Samples were collected at 0.25, 0.5, 0.75, 1, 1.5, 2, 4, 6, 8, 10, and 24 hr time points. Sampling was followed up with the dialysis buffer refreshments. In the end, the released Dox was determined with spectrofluorimetry (Shimadzu RF5000U, Japan) at 490 nm excitation and 585 nm emission (76).

***Animal studies***

*Ethical statements*

All animal experiments were conducted in compliance with the Institutional Ethical Committee and Research Advisory Committee of Mashhad University of Medical Sciences guidelines. For the biodistribution and pharmacokinetic studies, 8 to 10-week old healthy female NMRI mice were used. They were housed in standard cages with free access to water and food (standard laboratory rodent’s chow). The animal house temperature with a 12-hr light/dark cycle was maintained at 23.9/30.8 °C. All efforts were made to reduce the number of animals used and to minimize animal suffering.

*Biodistribution and pharmacokinetics*

In order to provide a technique with the highest encapsulation efficiency and stability, the formulations were prepared almost an hour before animal administrations. Briefly, the GSH-PEG prepared micelles were lyophilized and stored in sterile vials. Immediately before the administration, the separate components (micelle powders and the dextrose buffer) were mixed, solubilized, and post-inserted into the commercially available Caelyx^®^ nanoliposomes at 60 °C for 1 hr (77-79). The formulations were injected via the tail vein at 10 mg/kg of doxorubicin either as encapsulated in post-inserted preparations or Caelyx^®^. The controlled mice received dextrose 5%. Before euthanasia, mice were deeply anesthetized through a ketamine-xylazine cocktail by intraperitoneal injection. From all mice, blood samples were collected by heart puncture, and the whole brain, spleen, lungs, kidneys, heart, and a portion of the liver were dissected, weighed, and homogenized with a bead beater (Bead Beater, Biospec, Bartlesville, OK, USA) at 5,000 rpm. Regarding the blood serum collection, the blood was allowed to coagulate at 4 °C and followed with centrifugation for 10 min at 14,000 rpm. The serum (upper phase) was collected. The homogenized tumor samples and the sera were stored at 4 °C overnight to extract the drug. The samples were then centrifuged at 14,000 rpm and the supernatants were diluted and assessed at Ex: 490 nm, Em: 590 nm using a spectrofluorometer. The calibration curves were prepared using serial dilutions of the tumor and sera extracts of the control mice.

For the histological study, mice were injected IV by a dose of 10 mg/ml of each formulation (n = 3). 24 hr after injection, animals were euthanized, the brains of mice were removed and fixed. After embedding in paraffin, sectioning was performed and tissues were stained using fluroushiled™ with DAPI (Sigma-Aldrich). Then each section was photographed using fluorescent microscopy, and penetration of Dox into the brain tissue was determined. 

***Pharmacokinetic studies***

The blood samples were collected from each mouse at 24, 48, and 72 hr post-injection, and the Dox concentrations were assessed by spectrofluorimetry (Ex: 490 nm, Em: 580 nm). Considering the linear trapezoidal method and non-compartmental analysis for data obtained after intravenous bolus 10 mg/kg dose, the area under the concentration-time curve (AUC) and area under the first moment curve (AUMC) were calculated. Furthermore, the mean residence time (MRT) is obtainable via AUC and AUMC measurements. The elimination rate constant (Ke) and half-life (t_1/2_) plus the total clearance (Cl), and the volume of distribution (Vd) values were also determined.

***Statistical analysis***

Statistical analysis was conducted using GraphPad Prism version 6 (GraphPad Software, San Diego, CA). Two-way analysis of variance (ANOVA) plus Tukey’s *post-test* was used to find out significant differences between different groups. Data were considered significant when *P*<0.05.

## Results

***Characterization of glutathione targeted liposomes***

**Figure 1:**
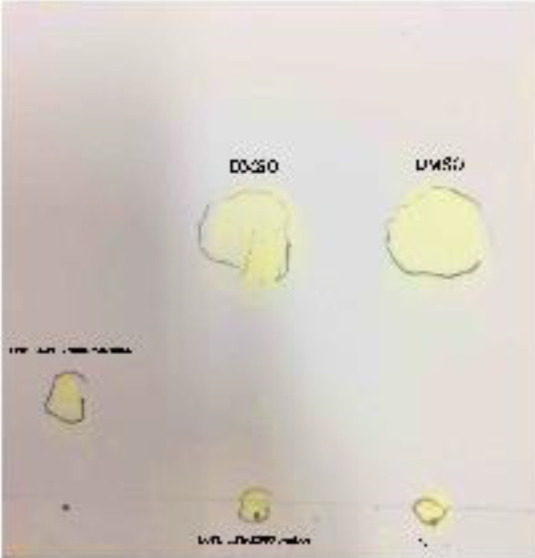
TLC chromatography. PEG and P+P are abbreviations for free PEG2000-MAL, PEG2000-GSH complex, and free GSH peptide, respectively. The chromatograph shows no similar spots in the (P+P) spot area which demonstrates the end of the conjugation reaction

The final product of conjugation was assessed by TLC and HPLC to confirm that the linking reaction was achieved efficiently. Before the GSH-PEG2000 complex incorporation into the liposomal structure, the peptide to lipid conjugation was assessed by TLC and HPLC (70). The unconjugated peptide and lipid plus the product of conjugation reaction due to different mobility on silica gel paper were confirmed with TLC (Figure 1). As expected, PEG and the GSH have traveled farther compared with the GSH-PEG2000 complex. No spots were found corresponding to the free PEG or the peptide migration distance suggesting a 100% linking efficacy in the conjugation reaction.

**Figure 2:**
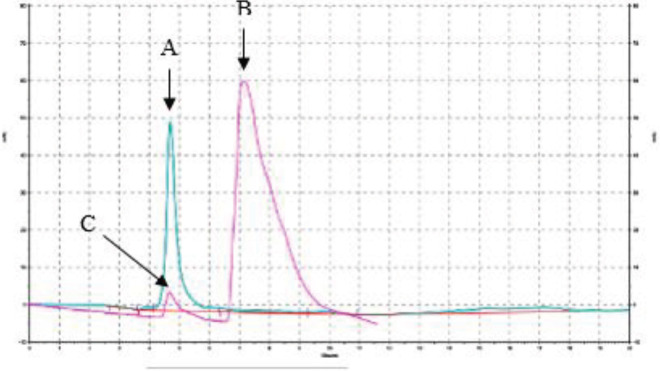
HPLC chromatography as the complex linking assay. (A) is the result of the first injection which represents the free peptide that eluted with a retention time of 4.7 min. (B) represents the PEG2000-GSH filtrate, and (C) represents the free peptide at the exact retention time as the free GSH which was injected to verify and identify the complex from the free peptide

 The free peptide and the final product’s HPLC chromatography based on the retention time are shown in Figure 2. At first, the free peptide was assessed, and 5 min post-injection a peak of the graph was made. Similarly, a conjugation final product was injected into the HPLC column and 7 min post-injection the complex graph was achieved. In the end, a mixture of free peptide and the complex were injected to verify and identify the presented curves (Figure 2).

***Liposomal characterization***

The post-insertion method was performed at 65 °C for 1 hr with gentle stirring of 25, 50, 100, 200, and 400 ligands PEG-GSH with the commercially available Caelyx^®^. The final liposomes were assessed for the drug remaining in liposomes after post-insertion by the fluorimetry method (Table 1). Moreover, the physical properties of the liposomal formulations including zeta potential, average size, and PDI were obtained by dynamic light scattering (DLS) (Table 1). Most of the formulation sizes were less than 100 nm which is preferable for nano-drug delivery through iv administration. PDI reports the uniformity of nanoparticles which is desirable (less than 0.2). The size of liposomal formulations was slightly increased by the number of the ligands as a confirmation of post-insertion. Although the size differences were not significant. Furthermore, the general particle size was negative due to the presence of DSPE-PEG(2000) Maleimide.

**Figure 3:**
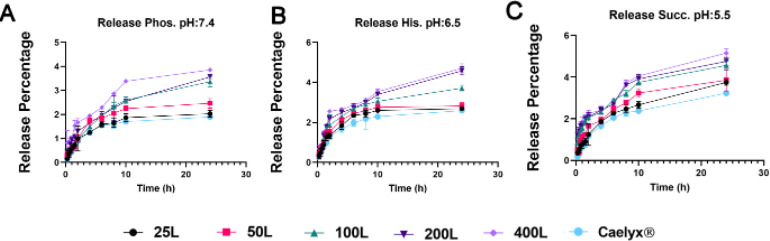
Release profile of different glutathione (GSH)-targeted nanoliposomal formulations at pHs of 7.4 (phosphate buffer), 6.5 (histidine buffer), and 5.5 (succinate buffer)

The release test as the leakage stability experiment was performed in different pH values of 5.5, 6.5, and 7.4 to simulate endosomal, tumoral, and physiological release (Figure 3). All of the formulations followed an increasing trend in presence of more acidic conditions. The release profile was 2–3 percent increased when formulated with the post-insertion method based on the number of the ligands compared with Caelyx^®^. Although none of the differences between modified and unmodified nanostructures were significant after 24 hr of release assessments.

***Biodistribution studies***

**Figure 4:**
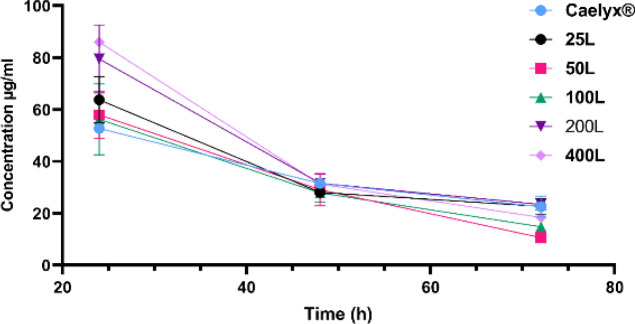
Blood distribution of glutathione (GSH) targeted nanoliposomes per milliliter serum after administration of an iv single 10 mg/kg dose of the formula. The blood serum values did not show any significant differences (*P*>0.05) between formulations. The level of Doxorubicin concentrations fell drastically over time

**Figure 5:**
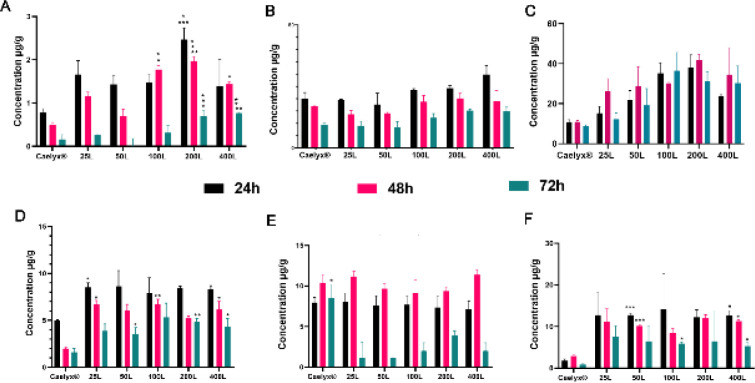
Caelyx and glutathione (GSH)-Caelyx formulations biodistribution at different time points (24, 48, and 72 hr post-injection) in (A) Brain, (B) Liver, (C) Spleen, (D) Heart, (E) Kidney, and (F) Lung in NMRI healthy mice after a single dose of GSH-Caelyx and Caelyx products i.v. administration with 10 mg/kg. Results are expressed as Mean ± SEM. One-way ANOVA was used to analyze the results. The meaningful differences between the treatment groups are shown as * (*P*≤0.05)

**Figure 6:**
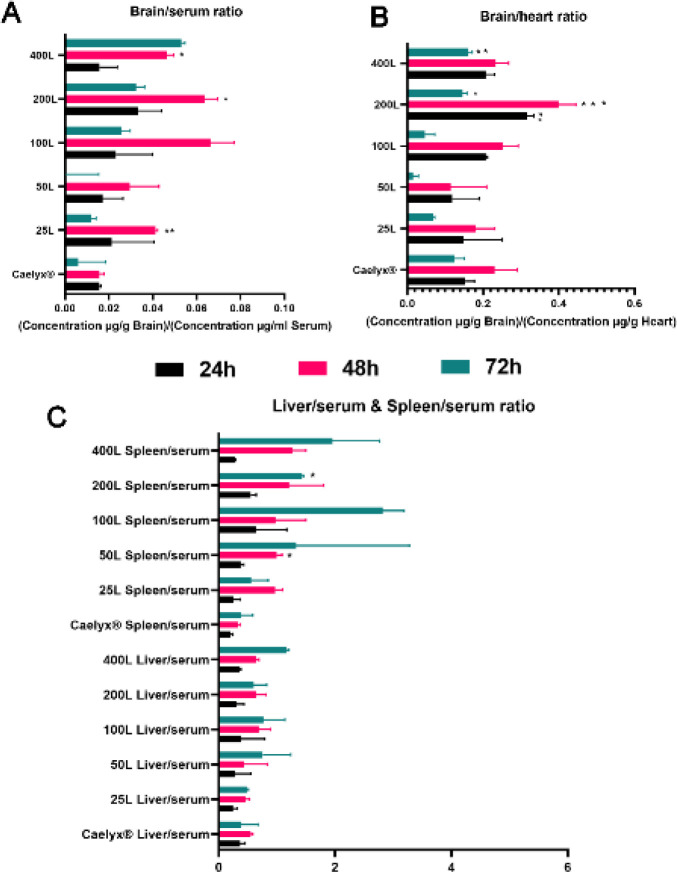
Panels (A, B, and C) indicate Doxorubicin concentration ratio in tissues to the concentration of Doxorubicin in each mouse serum. Panel A represents ratio of Dox concentrations in the brain to the micrograms of Dox per milliliter of serum. Panel B gives a comparison of Dox concentration between the brain and heart in each formulation to present an estimate of efficacy given by the formulations compared with their cardiotoxicity. Panel C illustrates ratio of Dox concentration in the liver/spleen tissues to the concentration of Dox in serum, respectively. Results are expressed as Mean ± SEM and the one-way ANOVA was performed to analyze the results

 In order to assess formulation distribution through the main organs (Brain, liver, spleen, kidney, lung, heart, and blood) we performed a comprehensive analysis of each tissue targeted liposome transportation, after 10 mg/kg doxorubicin formulation single-dose iv administration (Figures 4 and 5). It continued with ratio of brain, spleen, and liver tissue microgram distributed drug per gram tissue per milliliter serum. Additionally, the brain/heart ratio was assessed to provide further information concerning how effective they are to get to the brain besides cardiotoxicity as a doxorubicin serious side effect (Figure 6).

Based on the data represented in Figure 4, the blood serum after 24 hr did not show any significant differences (*P*>0.05) between formulations. The level of doxorubicin concentration was fallen drastically after 48 hr and continued downward after 72 hr. The formulations showed no significant differences in later time points. Generally, the serum concentrations declined gradually starting 24 hr after iv injection.

The analysis of whole-brain tissue (Figure 5-A) elucidated that all GSH post-inserted formulations had a higher level in the brain compared with Caelyx^®^. They had gradually decreased over time with no significant differences. However, over 24 hr post-administration, the 200L group showed a huge difference over Caelyx^®^. It also had a significant difference versus the 100L treatment group. The 100, 200, and 400 ligand formulations showed a markedly significant difference 48 hr post-injection. The 200L formulation had the highest concentration level in the brain tissue after 2 days. Moreover, a dramatic difference was detected between the 200L formulation and the Caelyx^®^ group. Its brain tissue doxorubicin levels were almost 2.8 and 1.7 folds greater than 50 and 25 ligand formulations, respectively. After 72 hr, the 200L and the 400L formulas showed better distribution over other treatment groups including Caelyx^®^, 25, and 50 ligands.

Even though the doxorubicin liver profile fell gradually over time, it was observed that its topmost concentration among treatment arms was dedicated to the 400 and 200 ligands at all-time points (Figure 5-B). On the contrary, the spleen accumulations did not follow a constant trend over time and were elevated after days 1 and 2 post-injection (Figure 5-C). However, they were slightly decreased after 72 hr. Noteworthy that the 400L and 200L spleen concentrations were significantly higher than Caelyx^®^ 24 and 48 hr after administration, respectively. None of the formulations showed any significant differences against each other in liver tissue except the 200L which had a meaningful drug content compared with the 50L after 48 hr. Furthermore, the 200L at its topmost concentration among treatment arms after 48 hr was found significantly more than the Caelyx^® ^group.

Generally, targeted treatment concentrations were higher than Caelyx^®^ as non-targeted nanoparticles based on the heart tissue analysis (Figure 5-D). Furthermore, the GSH targeted nanocarriers especially 25L, 100L, 200L, and 400L were observed with significant values over Caelyx^®^ at different time points. Based on the analysis of various tissues, there was no significant difference between the formulations in the kidney for 24 and 48 hr post-injection (Figure 5-E). However, the Caelyx^®^ level did not fall significantly in contrast with the targeted formulas after 72 hr which were drastically decreased. The glutathione-targeted nanoliposomes penetrated the lung tissue meaningfully compared with Caelyx^®^ with the least drug level at all-time points (Figure 5-F). Generally, the post-inserted formulas were accumulated in the heart and lung more than Caelyx^®^.

The presented analyzed data of brain/serum ratio (as a representative of the term brain-penetrant) elucidates that the post-inserted nanocarriers tend to accumulate and penetrate the brain tissue rather than circulate in the blood. The mentioned term quantitive numbers have appeared higher for 25, 200, and 400 GSH ligand nanoliposomes (Figure 6-A). Although the 24 and 72 hr time points do not show a noteworthy difference 48 hr past drug injection, the brain/blood ratio was found greater than 0.04 for 25,200 and 400 ligand formulas as the most brain-penetrants. At the same time point, the highest ratio was 0.06 for the 200L and 400L groups compared with 0.01 for Caelyx^®^. Figure 6-B shows that the targeted nanocarriers’ net accumulation in the brain is much more than their distribution in the heart which causes cardiotoxicity. The 200L had the highest brain/heart ratio at all times plus the 400L which had significant differences over other formulations especially the PEGylated liposomal doxorubicin (Caelyx^®^) at 72 hr. The liver/serum and spleen/serum ratios illustrate that the liver and spleen clearance of targeted treatments was increased over time and inconsistent with the number of GSH ligands (Figure 6-C). However, except for the 50L in 48 hr and 200L in 72 hr compared with Caelyx^®^, none of the elevations were found significant. 

**Figure 7:**
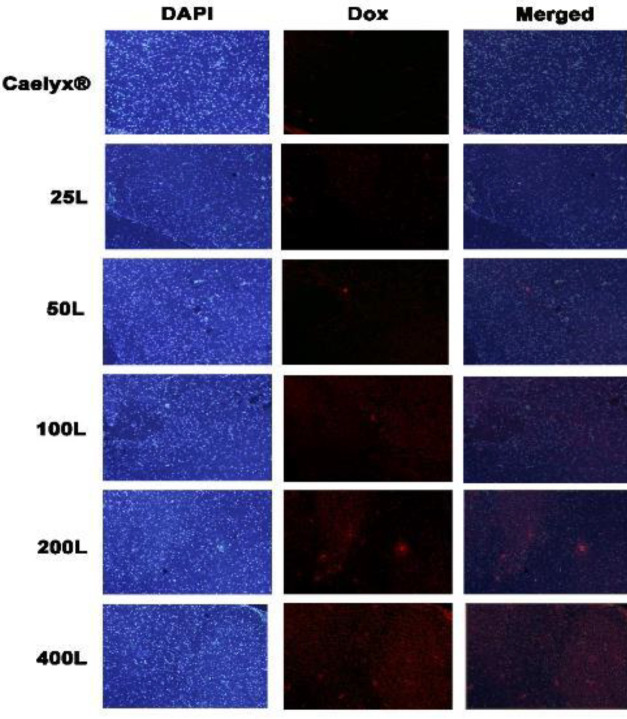
Fluorescent microscopy. Results of Dox internalization into the brain tissue are visualized by fluorescent microscopy. Staining the embedded sections was performed with DAPI. 200L and 400L showed higher internalization compared with Caelyx®. Sections are inspected under × 200 magnification

Figure 7 demonstrates the amounts of Dox penetration in the brain tissue based on the intrinsic fluorescence of the Dox. Qualification of the amounts of penetrated Dox using fluorescent microscopy shows higher accumulation of the 200L and 400L formulations compared with the others.

***Pharmacokinetic studies***

The given pharmacokinetic parameters in Table 2, present comparable values of AUC, AUMC, MRT, Ke, t1/2, and Vd between Caelyx^®^ and GSH-targeted formulations. The Caelyx had the least value for the AUC, as a representative of systemic exposure to treatment agent, compared with the targeted nanoliposomes such as 200 and 400 ligands.  The total area under the first moment curve (AUMC) was highest for the 200L and 400L nanoparticles. Accordingly, MRT as a result of AUC and AUMC values was found higher for the Caelyx treatment arm and was lessened inconsistent with increasing the number of the targeting ligands on the surface of the liposomes. The elimination rate and the volume of distribution were the least values for Caelyx. The half-life value which is the result of 0.693/Ke equation was the most for the non-targeted formulation (Caelyx). The obtained total clearance was achieved by dividing the dose by the AUC and was found to be minimum for the 200 and 400 targeted nanoliposomes. None of the proposed parameters had significant differences between each group.

TLC: thin-layer chromatography

**Table 1:** Physicochemical characteristics of the glutathione (GSH) targeted and Caelyx® formulations. Results are reported as (n = 3) mean ± standard deviation

**Liposomes**	**Z-Average Size** **(nm)**	**PDI**	**Zeta potential** **(mV)**	**Doxorubicin remaining in liposomes** **post-insertion** **(%)**
**25L**	94.6 ± 0.2	0.202	-19.2	96 ± 2.4
**50L**	93.87 ± 0.2	0.15	-22	95 ± 1.9
**100L**	94.56 ± 1.2	0.132	-20.1	99 ± 1.2
**200L**	96.5 ± 0.3	0.148	-20.1	97 ± 1.1
**400L**	102.4 ± 0.7	0.141	-21.7	96 ± 3.1
**Caelyx** ^®^	89.4 ± 1.8	0.127	-17	97 ± 2.4

**Table 2:** Pharmacokinetic parameters using non-compartmental methods in healthy NMRI mice following intravenous injection of Caelyx® and glutathione (GSH) targeted PEGylated nanoliposomal formulations (25, 50, 100, 200, and 400 GSH ligands) at a single bolus dose of 10 mg/kg

**Formulation**	AUC 0-t (μg/ml*h)	**AUMC 0-t (** **μ** g/ml*h^2)	**MRT 0-t (h)**	**Ke (1/h)**	**t1/2 (h)**	**Vd (l/kg)**	**Cl (l/kg h)**
**Caelyx**	3398.641761	88442.88	26.02300749	0.015621195	44.37222465	0.127990422	0.001999363
**25L **	4157.302675	87168.89664	20.96765703	0.022642377	30.61282807	0.086610597	0.00196107
**50L **	3884.688147	78742.64544	20.2700043	0.035684652	19.42423793	0.066774201	0.002382814
**100L **	4083.79918	82025.99328	20.08570688	0.028991073	23.90898704	0.074581266	0.002162191
**200L**	5567.938793	105391.7549	18.92832497	0.024485698	28.30824632	0.06188123	0.001515205
**400L **	5824.408664	98004.36096	16.82649117	0.037073224	18.69670654	0.043397168	0.001608873

## Discussion

In this body of research, we have determined the least possible GSH ligand effect to pass through the BBB in healthy mice. Moreover, the post-insertion method to incorporate targeting ligands into the liposomal structure was investigated. More precisely, the main objectives were applying and assessing the 25, 50, 100, 200, and 400 GSH ligands’ efficacy besides the post-insertion method’s applicability as one of the most preferable, fast, and cost-effective procedures in novel targeted nano-drug delivery systems with fewer preparation challenges (56, 80).

Briefly, the first experiment was to conjugate the GSH and DSPE-PEG(2000) Maleimide before the post-insertion (81). Conjugation of DSPE-PEG(2000) Maleimide and GSH was confirmed by TLC and HPLC to confirm the attachment was done perfectly which is shown in Figures 1 and 2. Table 1 is obtained after the post-insertion application. It evaluates the size, negative charge, PDI, and liposomal Dox content after the targeting ligands incorporation. The doxorubicin content of Caelyx^®^ did not alter significantly due to post-insertion (82, 83). The targeted formula size was approximately 96 nm which is slightly more than Caelyx^®^. The larger size could be due to the presence of the hydrophilic GSH peptide on the surface of the liposomes. The PDI for all of them was less than 0.2 which signifies their uniform distribution (84, 85). As was expected, they had more negative charges than Caelyx^®^ as the result of the GSH negative charge. The alteration of the size and zeta potential could be a sign of GSH conjugation on the surface of the nanoliposomes (86, 87). However, none of the sizes and charges were found significant in different groups. Therefore, our targeted nanoparticles were desirably expected to be biocompatible to stay stealth to the reticuloendothelial system (RES), blood circulation, and drug delivery. We conclude later statements based on the literature that describe an optimum nanoparticle as hydrophilic and negatively charged with an average of 100 nm of diameter (88-90). The encapsulation efficacy was calculated after post insertion which was more than 95% for all of the formulations. The post-insertion based on the drug loading efficiency did not affect the encapsulation considerably.

The release test showed that the leakage was not compromised significantly and the formulations stayed almost stable. The release profiles at different pH values were slightly increased. The released doxorubicin was assessed in correlation with the number of targeting ligands and more acidic pH (75). 

The pre-admixture of contents was prepared on the same day of the *in vivo* experiments to ensure a fresh, more stable, and consistent formula with a higher encapsulation efficiency is injected. The active components including the market available Caelyx and lyophilized GSH-PEG were mixed, measured, and linked immediately before drug administrations (91-94).

The *in vivo* experiments were done on non-tumoral healthy mice to evaluate the performance of GSH as a targeting ligand for the BBB. This experimental setting was used to demonstrate the physiological response. It is established that tumors can cause inflammation thus affecting tight junctions and permeability of the BBB and consequently facilitating the EPR effect to reach brain tissue (27, 95). Our investigation showed that the Dox serum level was increased gradually by the number of GSH ligands on the surface of the liposomes, but there was no significant difference between them. Nevertheless, the concentrations went drastically downward after 48 hr which could be due to drug distribution into the other tissues especially the brain, as the aim of this study, plus liver and spleen. The 200 and 400 ligand formulas were found at their highest level in the liver and spleen which is due to the RES uptake. Studies show that the elevation of the size of the formulations is associated with harvest of formulations (96, 97). The GSH negative charge could be another reason for the RES elevation, as Table 1 presents more negative charges on the formulations. It is worth mentioning elevated distribution of 200L and 400L to the other tissues plus increased RES did not affect the brain tissue drug delivery even without the EPR effect. The most accumulated nanoparticle in the brain was after 24 hr for the 200L. The 200L also showed higher levels of Dox even after 48 and 72 hr of iv injection. Based on the scrutinized literature, the idea that GSH could be one of the most successful targeting ligands ever tested to pass through the BBB was established. Even though it was checked that the conventional incorporation of 5% GSH will improve the doxorubicin brain biodistribution, the concept that even a few GSH ligands as post-insertion, as a straightforward cost-effective method, might have any considerable effect to pass through the brain was hypothesized and tested. As is observed in Figure 5-A, almost all of the targeted formulations had higher levels compared with Caelyx^®^. The 200 and 400 ligands were found with a meaningful difference in almost all timepoints. We consider the 100, 200, and 400L formulations as representative of the best post-inserted formulations, brain-penetrant, as they have achieved almost a 0.06 ratio. Deemed term in early discovery explains that they have adequate brain penetration to be considered for future experiments (98, 99). Additionally, the histological images indicate the acceptable penetration of 200 and 400 ligand formulations into the brain tissue the same as what was discussed earlier. The heart distribution of the targeted formulations was mainly more than Caelyx^®^. The higher heart distribution might be due to the higher GSH receptors on the heart tissue cells. As the heart accumulation of Caelyx^® ^is remembered as its main drawback, the insertion of GSH might be considered out of scope for brain drug delivery as for the cardiotoxicity. We challenged its cardiotoxicity by gaining a microgram of doxorubicin in brain cells. Therefore, the brain/heart ratio was investigated to evaluate how significant can GSH targeted formulations, as a measure of effectiveness, can get through the BBB even with the cardiotoxicity. The 200 and 400 ligands effectively overcame the heart accumulated toxicities by presenting optimum brain penetration and efficacy. Moreover, the lung accumulation of the post-inserted formulations was found significantly higher than Caelyx^® ^which could be explainable by the reported GSH receptors in the lung tissue. The pharmacokinetic profile of Caelyx^®^ and the GSH targeted nanostructures have been shown in Table 2. Mainly the targeted formulations, especially the 200L and 400L, had greater total drug exposures across time than Caelyx^®^. The Caelyx’s mean residence time was the highest among treatment groups which is mostly due to lower AUC value. Higher AUC for a given dose is correlated with lower clearance. The clearance concept comes from the distribution and elimination functions (100). The Ke was higher for all of the GSH targeted formulations and as a result, their time to reach a 50% decrease in plasma concentration or t1/2 was less than Caelyx^®^. This is reasonable with the resulting MRT. It is noteworthy to mention that higher Ke for GSH targeted formulations might be related to the tissue accumulations especially the brain which is desirable. As was discussed earlier, the GSH as an endogenous anti-oxidant is expressed on several issues. Therefore, the poor Vd represents that the drug’s propensity to remain in plasma is low and tends to redistribute in the non-specific tissues. The lowered clearance for 200L and 400L is the result of higher elimination constants. Additionally, due to the larger size and more negative charge of the nanoparticles, the RES can detect and be exposed to the targeted formulations and tends to have a higher elimination constant.

## Conclusion

This study evaluated the use of a post-insertion approach to target PEGylated liposomal doxorubicin nanoparticles to the brain. This method is more efficient, follows a simpler method of preparation compared with the pre-insertion approach (62, 101), and is likely more economical warranting a need for further cost-effectiveness analyses in clinical manufacturing processes. Interestingly, this approach led to increasing brain biodistribution for 200 and 400 ligands, suggesting that using more glutathione ligands can improve the concentration of accumulated doxorubicin and increase brain-penetrant dosage forms. GSH receptors are present on other tissues raising the possibility of observing toxicities. The evaluated brain/heart biodistribution ratio as an example in this study demonstrates the importance of identifying the therapeutic index for any future drug development. It would be really interesting to compare the efficacy and biodistribution of 2B3-101 with post-inserted formulations once the drug becomes commercially available for purchase. Another area of investigation would be to assess higher than 400 targeting ligands on the surface of nanoliposomes impact on crossing through the blood-brain barrier.

## Authors’ Contributions

MRJ, AB Study conception and design; AM, RVGh, MM, SShS Data processing, collection, and perform experiment; AM, RVGh, MM Analysis and interpretation of results; AM, SShS Draft manuscript preparation, visualization; LA, SHA, SAM Critical revision and editing of the article; LA, SHA, SAM, MRJ Final approval of the version to be published; MRJ, SAM, AB Supervision, funding acquisition.

## Ethics and Dissemination

This paper is part of the Ph.D. thesis of Amin Mehrabian (Grant number: 960833) supported by Nanotechnology Research Center, Mashhad University of Medical Sciences (MUMS), Mashhad, Iran. 

## Conflicts of Interest

The authors declare that they have no known competing financial interests or personal relationships that could have appeared to influence the work reported in this paper.
